# Differences in Recycling of Apolipoprotein E3 and E4—LDL Receptor Complexes—A Mechanistic Hypothesis

**DOI:** 10.3390/ijms22095030

**Published:** 2021-05-10

**Authors:** Meewhi Kim, Ilya Bezprozvanny

**Affiliations:** 1 Department of Physiology, UT Southwestern Medical Center, Dallas, TX 75390, USA; 2 Laboratory of Molecular Neurodegeneration, Peter the Great St. Petersburg State Polytechnic University, 195251 St. Petersburg, Russia

**Keywords:** ApoE, LDL receptor, endosome, Alzheimer’s disease, modelling, protonation, charged interaction

## Abstract

Apolipoprotein E (ApoE) is a protein that plays an important role in the transport of fatty acids and cholesterol and in cellular signaling. On the surface of the cells, ApoE lipoparticles bind to low density lipoprotein receptors (LDLR) that mediate the uptake of the lipids and downstream signaling events. There are three alleles of the human *ApoE* gene. Presence of ApoE4 allele is a major risk factor for developing Alzheimer’s disease (AD) and other disorders late in life, but the mechanisms responsible for biological differences between different ApoE isoforms are not well understood. We here propose that the differences between ApoE isoforms can be explained by differences in the pH-dependence of the association between ApoE3 and ApoE4 isoforms and LDL-A repeats of LDLR. As a result, the following endocytosis ApoE3-associated LDLRs are recycled back to the plasma membrane but ApoE4-containing LDLR complexes are trapped in late endosomes and targeted for degradation. The proposed mechanism is predicted to lead to a reduction in steady-state surface levels of LDLRs and impaired cellular signaling in ApoE4-expressing cells. We hope that this proposal will stimulate experimental research in this direction that allows the testing of our hypothesis.

## 1. Introduction

Apolipoprotein E (ApoE) is a protein that plays an important role in the transport of fatty acids and cholesterol and in cellular signaling [1,2,3,4]. On the surface of the cells, ApoE lipoparticles bind to Low Density Lipoprotein receptors (LDLR) that mediate the uptake of the lipids and downstream signaling events [1,2,3,4]. There are three alleles of the human *ApoE* gene—E2, E3, and E4—with two of these alleles co-expressed in many tissues, including the central nervous system [4,5,6,7,8]. These alleles differ in amino acids in positions 112 and 158 of N-terminal ligand domains as follows—E2 (C112, C158), E3(C112, R158) and E4(R112, R158). There is well established genetic association between the presence of different alleles of the *ApoE* gene and human diseases [9,10,11,12,13]. In particular, the presence of ApoE4 is a major risk factor for developing Alzheimer’s disease (AD) late in life [14,15,16,17,18,19,20]. The mechanisms responsible for biological differences between different ApoE isoforms are not well understood and are currently under intense investigation [21,22,23].

**Figure 1:**
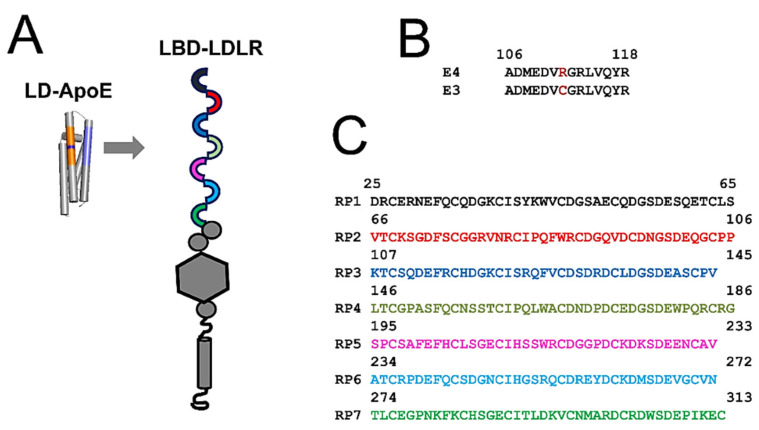
Ligand domain (LD) of ApoE and LDL-A repeats of LDLR. (**A**) The secondary structure of ApoE-LD and the domain structure of LDLR are shown. The receptor-binding interface is a part of the fourth α-helix in the ApoE-LD domain (shown in blue). The R112C domain portion of the third α-helix in the ApoE-LD domain is shown in orange. Position 112 within the R112C domain is shown in purple. Seven amino-terminal LDL-A repeats are shown as colored semicircles. (**B**) Primary sequence alignment of R112C domains from ApoE4 and ApoE3 isoforms. Amino acid in position 112 is shown in red (R for ApoE4 and C for ApoE3). (**C**) Primary sequences of seven LDL-A repeats from the LDLR. The sequences of RP1–RP7 repeats are color-coded according to the panel A diagram.

The ApoE protein is composed of the amino-terminal (N) and carboxy-terminal (C) domains, which are loosely hinged to each other [24,25,26]. The N-terminal ligand domain (LD) is involved in the association with surface receptors including LDLR (Figure 1A, Supplementary Figure S1); the C-terminal domain is involved in the binding of cholesterol and fatty acids [27,28,29]. Both domains form α-helical bundles [24] with the critical allelic positions C112 and C158, which are located on the side of the fourth and third α-helixes in the ApoE-LD domain.

LDLR is a single transmembrane domain protein that contains seven class A repeats (LDL-A) on its amino terminal (Figure 1A, Supplementary Figure S1) [30]. These repeats are approximately 40 amino acids long and constrained by three internal disulphide bridges that contain conserved DCXDXSDE motifs [31] (Supplementary Figure S1B). These domains are involved in the association with extracellular ligands [2,6,32]. The cytosolic tail of LDLR mediates signal transduction and endocytosis [5,33,34,35]. Similar to other LDLR ligands, ApoE lipoparticles bind to LDL-A amino-terminal repeats [36,37,38,39,40,41] (Figure 1A, Supplementary Figure S1). A ligand binding to the LDLR triggers cellular signaling transduction and the internalization of the ligand–receptor complex via clathrin-assisted endocytosis [1]. The internalized complex is sorted to endosomal compartments where the ligand is released and LDLR is recycled to the plasma membrane [42,43,44]. Multiple sources of evidence demonstrate that LDLR recycling requires organized cellular actions including luminal endosomal acidification and calcium (Ca^2+^) signaling [45,46,47,48,49,50,51]. In particular, endosomal acidification plays a key role in the control of LDLR recycling [40,52,53].

Interestingly, experimental evidence suggests that there are significant differences in the recycling of LDLR complexed with ApoE3 or ApoE4 [54,55,56,57]. However, a mechanistic explanation for the differences in ApoE3- and ApoE4-containing complexes is lacking. To explain these findings, we propose that the observed differences are due to the differences in the pH-dependence of the association between ApoE3 and ApoE4 isoforms and LDL-A repeats of the LDLR. As a result, ApoE4-containing but not ApoE3-containing LDLR complexes are trapped in late endosomes and targeted for degradation, leading to a reduction in the steady state surface levels of LDLRs and impaired cellular signaling in ApoE4-expressing cells.

## 2. Results

### Estimated Association Energy between ApoE3/4 and LDL-A Repeats of LDLR

The N-terminal LD domain of ApoE is folded into a bundle of four α-helixes (Figure 1A, Supplementary Figure S1). The portion of the fourth α-helix contains Lysine- and Arginine-rich region (labeled blue in Figure 1A and Supplementary Figure S1) which is directly involved in the association with LDLR and other ApoE receptors [24,25,26,36,37,38,39,40,41]. The single amino-acid difference between ApoE3 and ApoE4 isoforms at position 112 (labeled purple in Figure 1A) is located within the third α-helix of the ApoE domain. A portion of the third α-helix is shown by the orange color in Figure 1A and Supplementary Figure S1 and the corresponding sequence is shown in Figure 1B. This portion of the third α-helix is selected because it is located parallel to a portion of the fourth α-helix that is directly involved in the association with LDLR (Figure 1A, Supplementary Figure S1). We named this portion of the third α-helix the “R112C domain” and its sequence is shown in Figure 1B. ApoE3 contains Cysteine and ApoE4 contains Arginine in position 112 (Figure 1B). As is the case with ApoE3, ApoE2 contains Cysteine in position 112 and, for the purposes of our study, we focused on ApoE3 only. We reasoned that the Arginine is more likely to be protonated by the acidification inside endosomal compartments than Cysteine, leading to differences in the local charge of the third α-helix between the ApoE3 and ApoE4 isoforms. The third α-helix of ApoE-LD is located in close proximity to the binding interface between ApoE and LDLR (Figure 1A, Supplementary Figure S1). Thus, we reasoned that the changes in charge of the R112C domain may exert different electrostatic effects on the association between the ApoE-LD and LDL-A repeats of LDLR.

**Figure 2:**
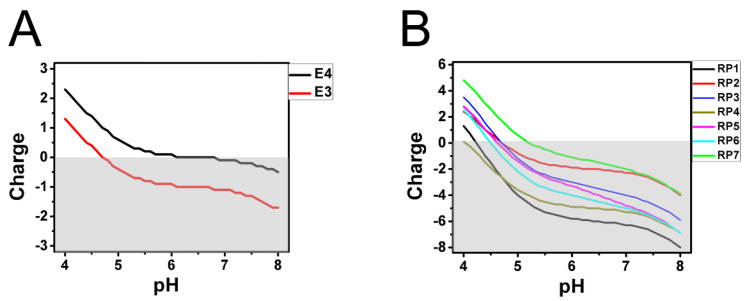
Local charge calculations for the R112C domains of ApoE3/4 and LDL−A repeats. (**A**) Local charges of R112C domain from ApoE3 (red line) and ApoE 4 (black line) were calculated as a function of pH in the range between 4 and 8. (**B**) Local charges of each of seven LDL−A domains were calculated as a function of pH in the range between 4 and 8. The charge value curve for each LDL−A repeat is color-coded as on panel 1A.

To test this hypothesis, we calculated the local charges of the ApoE3 and ApoE4 R112C domains as a function of pH in the range between 4 and 8. We also performed similar calculations for each of the seven LDL-A repeats (Figure 1C). These calculations predicted gradual charge shift from a negative charge at pH 8 to positive charge at pH 4 for these domains (Figure 2). At each pH value, the local charge of the R112C domain from ApoE4 was more positive than the local charge of the R112C domain from ApoE3 (Figure 2A). With some variability among LDL-A repeats, at each pH value the charge of the seventh repeat (RP7) was the most positive and the charge of the first repeat (RP1) was the most negative (with the exception of pH values below 4.6, where the fourth repeat, RP4, became the most negative) (Figure 2B).

**Figure 3:**
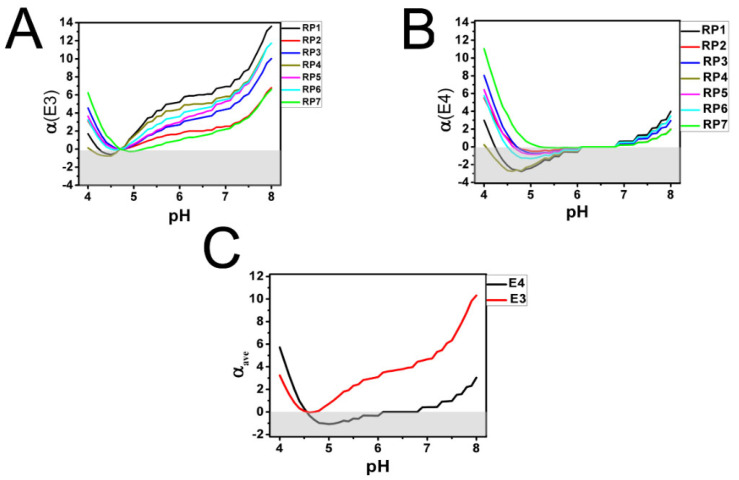
The product of ionic charges (α) of electrostatic interactions between R112C domains of ApoE3/4 and LDL−A repeats (**A**,**B**). The product of ionic charges (α) of the R112C domain from ApoE3 (**A**) and ApoE4 (**B**) and each of the seven LDL−A repeats is shown as a function of pH in the range between 4 and 8. The values of α for each LDL-A repeat are color coded as on panel 1A. (**C**) Average value of α for all LDL−A repeats are shown as a function of pH in the range between 4 and 8 for ApoE3 (red line) and ApoE4 (black line).

The energy of electrostatic interactions between the R112C domain of ApoE and each of the LDL-A repeats of LDLR is governed by Coulomb’ law, which states that the interaction energy is proportional to the product of these local charges. The positive product represents a repulsive electrostatic effect, and the negative product represents an attractive electrostatic effect. In the first approximation, we considered the R112C domain and each of the LDL-A repeats as point charges and calculated the product of these local charges (α) as a function of pH. The calculated values of α for the ApoE3 and ApoE4 R112C domains are shown in Figure 3A,B. In the case of ApoE3 at each pH value, the α was either positive or close to 0 at pH values near 4.75 (Figure 3A). Only for the first repeat, RP1, was the α slightly negative in the range between 4.25 and 4.75 (Figure 3A). In contrast, for ApoE4, the α was negative for most LDL-A repeats in the range of pH between 4.5 and 6 (Figure 3B). To estimate combined effect on association between the R112C domains and LDL-A repeats, we averaged the values of α across all seven repeats. The average interaction curves (Figure 3C) suggest strong repulsive interactions between the R112C domain of ApoE3 and LDL-A repeats in the range of pH between 5 and 8, and between 4 and 4.5 (Figure 3C), and minimal interactions in the range of pH between 4.5 and 5 (Figure 3C). In contrast, there is a weak repulsive interaction between the R112C domain of ApoE4 and LDL-A repeats in the range of pH between 7 and 8, minimal interaction in the pH range between 6 and 7, and attraction in the pH range between 4.5 and 6 (Figure 3C). Typical physiological pH outside of the cells is close to 7.5 [44,49,58,59]. The range of pH values inside endocytic compartments is between 5.5 for late endosomes (LE) and 6.5 for early endosomes (EE) [47,51,60,61]. There is a significant difference in electrostatic interaction energies between R112C domains from ApoE3 and ApoE4 and LDL-A repeats in the pH range between 4.5 and 7.5 (Figure 3C). We propose that this difference may contribute to biological differences between these isoforms. 

## 3. Discussion

### Endocytosis and Recycling of ApoE3 and ApoE4-Containing LDLR Complexes

**Figure 4:**
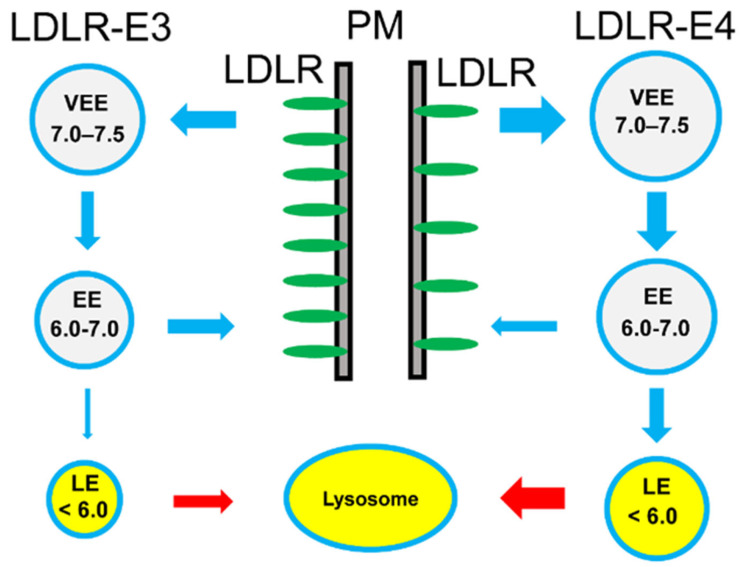
Endocytosis and recycling of ApoE3 and ApoE4-containing LDLR complexes. Surface LDLR (R) form complexes with ApoE3 (E3) and ApoE4 (E4) at plasma membrane (PM). Following internalization after clathrin-mediated endocytosis, these complexes are moved from the very early endosomal (VEE) compartment to the endosomal compartment (EE) and the late endosomal compartment (LE). Dissociation of ApoE allows recycling of LDLR to the plasma membrane. The LDLR trapped in LE compartments are targeted for degradation by lysosomes. The range of pH values for each endosomal compartment is indicated.

Expression of ApoE4 allele is a strong risk factor for Alzheimer’s disease (AD) and for many other disorders of aging [15,18,62]. In contrast, expression of the ApoE3 allele does not have such an effect. There is experimental evidence that defects in the recycling of ApoE4 and its receptors could contribute to brain dysfunction in AD patients [55,63,64,65]. Based on our modeling results (Figure 3), we propose a mechanistic explanation for the differences in recycling of ApoE3-containing and ApoE4-containing receptor complexes (Figure 4). Specifically, we propose that both ApoE3 and ApoE4 bind LDLR on the surface of the cell. At pH values of 7.5 in the extracellular media, we expect that the affinity of the ApoE3 isoform for LDLR will be slightly lower than the affinity of the ApoE4 isoform due to stronger electrostatic repulsion between the R112C domain and LDL-A repeats (Figure 3C). This prediction is consistent with experimental studies of ApoE3 and ApoE4 association with LDLR [63,64,66,67]. Following internalization after clathrin-mediated endocytosis, ApoE3-containing and ApoE4-containing LDLR complexes move to very early endosomal (VEE) compartments (Figure 4). The pH values inside these compartments are in the range between 7.0 and 7.5 [47,51,60,61], which is similar to extracellular media. From VEE compartments, ApoE-LDLR complexes proceed to early endosomal compartments (EE) with intraluminal pH in the range between 6 and 7 [47,51,60,61]. At these pH values, our model predicts significant electrostatic repulsion between the R112C domain of ApoE3 and LDL-A repeats, but minimal electrostatic interactions between the R112C domain of ApoE4 and LDL-A repeats (Figure 3C). Thus, LDLRs are able to easily dissociate from the ApoE3 complex inside EE and recycle back to the plasma membrane (Figure 4). In contrast, a significant fraction of LDLRs remain bound to ApoE4 and trapped in EE compartments (Figure 4). Remaining complexes of LDLR and ApoE3/4 move to the late endosomal compartment (LE) (Figure 4) with intraluminal pH in the range of 5.0–6.0 [47,51,60,61]. At these pH values, our model predicts electrostatic repulsion between the R112C domain of ApoE3 and LDL-A but electrostatic attraction between the R112C domain of ApoE4 and LDL-A repeats (Figure 3C). Therefore, inside LE, remaining LDLR dissociate from ApoE3 complexes for recycling to the plasma membrane, but are trapped in ApoE4 complexes and targeted to lysosomes for degradation (Figure 4). Due to defects in recycling, our model predicts that steady state levels of LDLR on the plasma membrane surface are lower in aging ApoE4-expressing cells than in ApoE3-expressing cells (Figure 4). Although not modelled in this study, it is likely that similar conclusions can be reached regarding other types of ApoE receptors in addition to LDLR.

Even among patients that express the ApoE4 isoform, there is a significant variability in developing AD and in the rate of disease progression. It is possible that this variability can be attributed to the differences in sex and in life-long environmental stresses and factors [68,69]. Other factors that could affect ApoE-LDLR recycling are related to posttranslational protein modification. For example, effects of O- or N-glycosylation of LDLR have been shown to affect its ligand affinity [70,71,72]. Additional modelling studies will be required to incorporate the potential effects of these modifications of LDLR on the association with ApoE3 and ApoE4.

In summary, we propose that the reduction in levels of LDLR and other ApoE-binding receptors at the plasma membrane (Figure 4) leads to impaired lipid metabolism and cellular signaling and predisposes these neurons to disorders of aging and AD. We hope that this proposal will stimulate experimental research in this direction that allows the testing of our hypothesis.

## 4. Materials and Methods

Crystal structures of the ApoE N-terminal LD region (pdb 1B68 and 1NFN) [21,73] and the extracellular domain of LDLR (pdb 1N7D and 1AJJ) [74,75] were downloaded from PDB bank and analyzed using Coot (Crystallographic Object-Oriented Toolkit, v0.8.9.2) [76] and Pymol (The PyMOL Molecular Graphics System, Version 2.0 Schrödinger, LLC) programs. A publicly available calculator program (PROTEIN CALCULATOR v3.4, http://protcalc.sourceforge.net/, accessed on 1 December 2020) was used to estimate charges. The graphs and Figures were produced using Origin v9.0 (OriginLab Corporation, Northampton, MA, USA).

## Data Availability

Not applicable.

## Supplementary Materials

The following are available online at https://www.mdpi.com/article/10.3390/ijms22095030/s1, Figure S1. Structures of ligand domain (LD) of ApoE and LDL-A repeats of LDLR.
