# Supplementary material for: Differences in Recycling of Apolipoprotein E3 and E4—LDL Receptor Complexes—A Mechanistic Hypothesis
Source: Int J Mol Sci. 2021 May 10;22(9):5030. doi: 10.3390/ijms22095030 (PMC8126166; doi:10.3390/ijms22095030)
Supplement: Supplementary file 1 [file ijms-22-05030-s001.zip › ijms-1216730-supplementary.pdf]

## Supplementary Materials

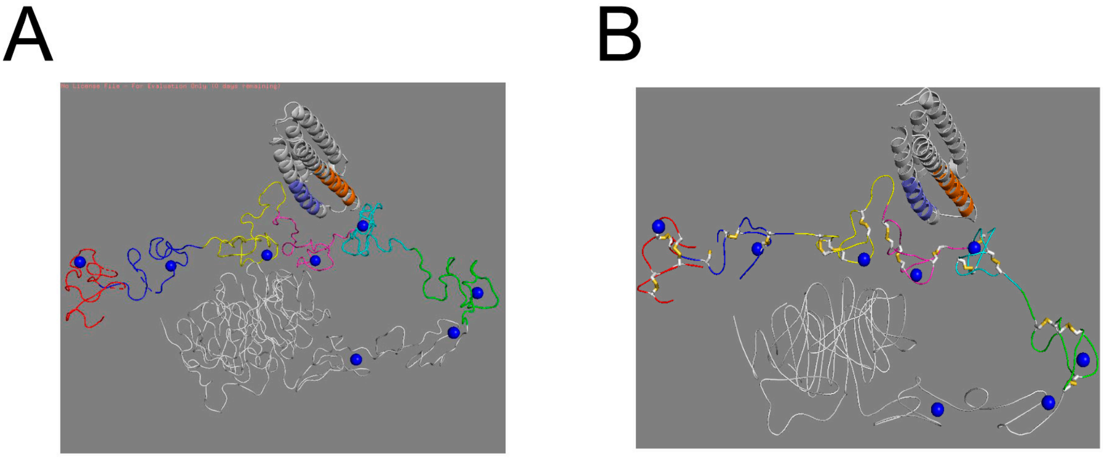


**Figure S1.** Structures of ligand domain (LD) of ApoE and LDL-A repeats of LDLR.
